# Light people: Prof. Evelyn Hu’s adventure: from semiconductors to quantum – from industry to academia

**DOI:** 10.1038/s41377-023-01192-5

**Published:** 2023-06-26

**Authors:** Yating Wan, Chenzi Guo

**Affiliations:** 1 Integrated Photonics lab, King Abdullah University of Science and Technology, Thuwal, Makkah Province Saudi Arabia; 2 Changchun Institute of Optics, Fine Mechanics and Physics, Chinese Academy of Sciences, Changchun, China

**Keywords:** Quantum optics, Optoelectronic devices and components, Nanocavities

## Abstract

As part of our Light People series, we are delighted to have invited Prof. Evelyn Hu, a highly accomplished scientist from Harvard University, to share with us her personal journey. Prof. Hu’s remarkable contributions in both academia and industry have taken her from industry giants to academia’s most prestigious institutions, traversing various research frontiers that have played a critical role in the ongoing digital revolution. Through this interview, we aim to offer the Light community valuable insights into nanophotonics, quantum engineering, and Prof. Hu’s research methodology and life philosophy, while also celebrating her outstanding achievements as a female role model. Ultimately, our goal is to inspire more women to pursue careers in this important and rapidly expanding field, which has a profound impact on all sectors of society. The following is a summary of an interview with Professor Evelyn Hu, available in the Supplementary Information.

Prof. Evelyn Hu is a distinguished physicist and materials scientist at Harvard University who has made significant contributions to the field. She was mentored by Prof. Chien-Shiung Wu, a well-known physicist often called the “First Lady of Physics” and “Chinese Marie Curie.” Prof. Hu is a highly respected academic and has been elected to several prestigious academies and fellowships, including the National Academy of Sciences, National Academy of Engineering, American Academy of Arts and Sciences, IEEE, APS, and AAAS.

**Figure:**
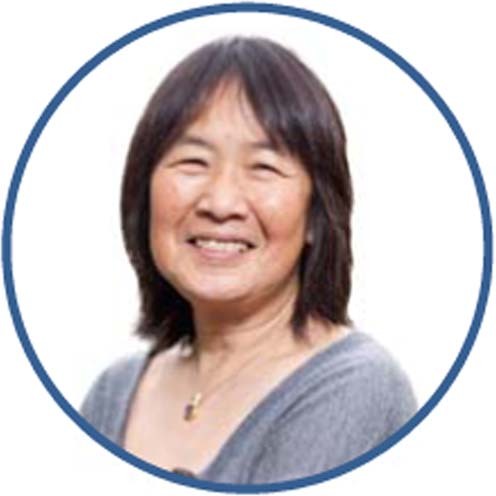

Previously, Prof. Hu worked at AT&T’s Bell Laboratories and was a full professor at the University of California, Santa Barbara. She was also vice-chair and chair of the Department of Electrical and Computer Engineering at UCSB and served as scientific co-director of the California NanoSystems Institute. Prof. Hu is a pioneer in nanofabrication research, focusing on high-resolution patterning and etching of circuits on nanoscale materials and developing biological approaches to nanotechnology. She co-founded Cambrios Technology, which focused on developing cost-effective materials for electronic devices (the successor company in Cambrios Advanced Materials). Prof. Hu has received many awards and honors, including the NSF Distinguished Teaching Fellow award and the AAAS Lifetime Mentor Award, and holds honorary doctorates from the University of Glasgow, Heriot Watt University, Hong Kong University of Science and Technology, Notre Dame University, ETH Zurich, and City University of Hong Kong.

**Q1: Your influential work in nanofabrication has included high-resolution patterning and high-resolution etching of circuits onto nanoscale materials. How did you move into this research direction?**

**A1:** Briefly speaking, simply, and very fortunately, by following my interests. More specifically, it started from my first job at Bell Labs. My graduate studies were actually related to accelerator physics and particle physics, which were very different from what I’m doing now. The change in fields occurred when I went to Bell Labs, making it important that I learn a great deal of new techniques and disciplines from the very beginning. But I was fortunate enough to work with a group of people who are still my colleagues and friends, and we collectively became interested in developing the tools to make structures smaller. We had and have always believed, that when you are capable of sculpting nanostructures into a material, many of their physical behaviors can be profoundly different than what you can observe in the bulk material. Therefore, it’s the enticing promise of finding something different that started me on a lifelong interest in nanoscale etching, fine lithography, and techniques to use and understand the material so that we could create small structures of nano-sculpted materials that would behave differently.

**Q2: In a very short time, some of your research ideas have led to your co-founding of Cambrios Technology. Could you give us an introduction of this startup? What was the initial motive, and what was the progress?**

**A2:** Cambrios is part of the excitement, rewards and joy of working in academia. Dr. Angela Belcher did her Ph.D. at UCSB. She had a strong background in materials and biology. She chose to work with me as a post-doc, and it was really some of her seminal insights that led to the idea of using bio-templating to create artificial materials with a nanoscale pattern that was defined by the biological entity. This powerful idea drove the inception and founding of Cambrios. Though what Cambrios eventually focused on is very different, the bio-templating ideas gave us an insight into a new way to make large-scale transparent conductors that could be conformable and manufacturable in roll-to-roll quantities. So, it taught me a profound lesson about the benefits of adopting a flexible response to scientific opportunities and being alert to the fortunate, unintended consequences.

**Q3: Your current research focus is on photonics and material sciences towards energy and environmental technologies. Could you share your perspective on exploring the use of gallium nitride wafers at nanoscale levels and the use of quantum dots?**

**A3:** In addition to sculpting materials at the nanoscale to transfer information for devices and systems, I’ve always been intrigued by the possibilities offered by new materials to modulate, control, and utilize information in different ways. You mentioned gallium nitride, on which the 2014 Nobel Prize was given. Gallium nitride was not a brand-new material that people had never imagined to be possible. In fact, researchers had thought for a long time that gallium nitride could be a new material of great importance. But it was Shuji Nakamura and his colleagues that rose to the challenges of this very difficult material (which remains a difficult material) over a long period of time. What possibilities for science and technology can result from access to a new material? How does the world change if somebody suddenly find a way to synthesize a material that hasn’t been available before? You’ve seen what’s happened with gallium nitride, you’ve seen there is a revolution in lighting, you’ve seen the way we use displays, the way we transform information, the way LEDs decorates and illuminate our buildings and our environments; All this is made possible through inexpensive, low-dissipation light-emitting-diodes and lasers. Gallium nitride took us into a regime where we could not only complete the visible spectra by integrating sources of green and blue light, but also offered a possibility of light emitters in the UV region.

So, in a similar sense, I think new materials offer new possibilities of transforming science, technology, and our world. If you take a material and find a way of making it as quantum dots which are groups of a hundred or a thousand atoms then you have an artificial atom, a big atom that behaves as if it were a single atom. It has electronic and photonic capabilities that allow us to control the wavelength and lifetimes of the emission, which will enable more efficient optical devices.

On the other hand, while we both know that gallium nitride is a commercially important material, it still has a lot of strain and defects. But if we go to the other end of the spectrum and think about working with the defects themselves, as well as impurities in the materials, there’s a broad frontier of opportunities and challenges. That’s the focus of my research now. For instance, we take silicon carbide and remove a silicon atom to obtain a silicon vacancy; we take diamond, remove a carbon atom and put a substitutional nitrogen atom nearby to get a nitrogen-vacancy complex in the diamond. While we may have initially considered these defects as problems that we wanted to eliminate, it turned out that these defects have tremendously important properties. In addition to the light emission at different wavelengths, their photon signals are an indication of the net electron spin of these materials. In quantum mechanics, spin-up and spin-down states make up digital information, i.e., 0 s and 1 s. If we could control the spin state, if we could control the polarization of materials and create arrays where we have spin up and down, that is a rich source of information. The problem is how we can possibly control and read out the spin polarization of electrons within the usual material at room temperature? Part of the way to do that is through these special quantum color centers, where the optical signal is an indication of the spin polarization, and where we can use light to initialize a spin polarization and to read out a spin polarization. That’s exciting because it transforms our way of looking at nature: every part of nature - including the imperfections - is there for us to “play with”, to refine, and to use. Now that we begin to use this sort of quantum information, we can start to use the concepts of superposition and entanglement and enrich our ability to modulate information tremendously. So that’s the area I’m working in now, and there are many more possibilities and challenges for the future. For example, how do you create those defects? How do you control them? How do you understand them? How do you link them up into building blocks? How do you link the building blocks into systems?

**Q4: Following this question, do you think they can eventually find use in applications such as smartphones?**

**A4:** Absolutely. I think the application is happening today. As promising as quantum dots are and have been, many people still thought that the scaling up, the manufacturability, the lifetime, and the robustness would be issues. Yet, quantum dots are now being used in commercial television sets because of their capability to finely tune the color. To make the device perform better, we need to understand and control the materials so that they can withstand extreme temperatures with improved spectral purity, low resistivity, and low dissipation. All of this comes from the understanding of the materials that we already have and the new materials that people are constantly discovering.

**Q5: Could you share your perspective on using silicon carbide semiconductors for empowering battery electronic vehicles?**

**A5:** There’s a tremendous opportunity. It’s not a brand-new area, but much progress is needed in device operation at higher frequencies, higher powers, and higher temperatures. For power devices, I would like to defer to my longtime colleague at UCSB, Umesh Mishra, an expert in high-frequency and high-power electronic devices. He’s created a number of companies, including Transphorm, a pioneer in high-performance gallium nitride power conversion products. Like gallium nitride, silicon carbide is also a wide bandgap material, and you can get four-inch and six-inch wafers of different polytypes. Silicon carbide can also be used in electronic vehicles: there are opportunities that build on rapid switching and the rapid modulation possible with SiC devices. In addition, these wide bandgap materials can also find applications in space, LiDAR, long-distance sensing, and satellite communications that would need and welcome innovations in this regard.

**Q6: What do you expect to happen with the fourth-generation semiconductors? What do you think will be the next potential photonic materials after silicon carbide, gallium nitride, diamonds, etc.?**

**A6:** There’s been a lot of discussion about the next-generation semiconductors, and our understanding and definition of semiconductors has broadened tremendously. When speaking of semiconductors, most people would think about silicon, gallium arsenide and gallium nitride. But now we can synthesize a variety of materials that behave like semiconductors, i.e., materials with a bandgap, a conduction band, and a valence band, but with a broad variety of properties. Under certain circumstances, semiconductors can exhibit very different properties besides the typical the light-emission and conductivity properties. They also exhibit ferroelectric, ferromagnetic properties, etc., and there are different ways to control and to modulate. We’re beginning to utilize these unusual effects (electro-optical effects, electromagnetic, power-magnetic) and incorporate them into devices to achieve multifunctional devices. Such devices are neither purely optical (like a laser) nor purely electronic (like a transistor) but can be modulated and controlled in many ways. There is a whole world of newly synthesized semiconductors at our disposal. Some of the semiconductors are manifested in the so-called two-dimensional materials. One prototype of the two-dimensional materials is graphene, which was unusual itself with high electrical and thermal conductivities. But then, we have other two-dimensional materials, e.g., Transition Metal Dichalcogenides, Molybdenum disulfide, Tungsten disulfide, Tungsten diselenide. These materials do have a band gap but at the same time, can be a monolayer or several monolayers thick, which offers a tremendous advantage for their integration into devices. When stretching something with monolayers thick, the strain or deformation, in turn, changes the bandgap, and therefore, changes the behavior of these materials. Many of these semiconductor materials have displayed optical emission. They have long-lived, strongly bound excitons. People are just beginning to explore the potential of these two-dimensional materials for optical devices.

**Q7: You used to describe your research as “** ***playing with colors at the nanoscale*** **.” What are the major challenges and motivation?**

**A7:** By “*colors*,” I mean emission wavelengths that convey information. The more colors you have, the more information you have. That’s clear in optical displays because the more colors you have, the more you can modulate the brightness, the intensity, etc., and the more information you can convey. But beyond displays, the color is also information. Suppose you have narrow spectral width and have information that is transferred in parallel along with different colors. That represents a tremendous amount of information that you can convey simultaneously. The goal of our research on devices and on systems is to provide information, be it in a visual form or in an electronic form, for computation, for communications, and for everything in our lives.

By “*playing*”, I want to make the point that the world of research should be playful. It should be playful in a way that suggests that we enjoy research as we enjoy playing. We used our creativity to play with ideas of how to take materials and make them better as if we were playing games. By “*at the nanoscale*”, I underscore the unusual way that physical phenomena may change by changing the length scales of the materials. In summary, the whole idea of “*playing with colors at the nanoscale*” is basically to understand how we can take the materials in nature and use our creative ideas to sculpt those materials so that we can get more information and build more powerful information systems.

**Q8: You are now the co-director of the Harvard Quantum Initiative. What role will quantum play in your future research?**

**A8:** In fact, quantum has been my research focus for the past few decades. When I was still at UCSB, I began to explore quantum dots and quantum wells, which have uniquely defined characteristics and quantum mechanical behaviors. I worked with colleagues on ideas for creating a unique environment to obtain coupled light-matter interactions, e.g., the strong interactions between the electronic states of the emitter (like the quantum dots) and the photonic states of the cavity (like micro-disks, photonic crystal cavities). The experience there allowed me to move on from quantum dots in different materials to the color centers I work with now, and to address some more challenging problems: how to design cavities that will strongly couple with atom-sized emitters (the color centers); how to place those emitters within the right position in the cavities to create the coupling. Since the year 2000, I’ve been doing the precursors to support quantum information systems.

**Q9: What’s your blueprint for this Harvard Quantum Initiative?**

**A9:** It’s not my blueprint; I’ve been fortunate to work with an exceptional group of talented and dedicated colleagues who started some of the initial work before I joined Harvard. They are committed to various aspects of quantum: arrays of cold atoms, arrays of cold molecules, quantum materials, topologically protected materials, color centers in diamonds, etc. People enjoyed working collaboratively, worked together to bring further resources, and their students naturally interacted with each other. Thus, an informal, but enduring community was created. This provided the basis and motivation for seeking to create a more enduring and sustainable structure. In 2018, we were granted some seed funds to form the Harvard Quantum Initiative. The seed fund allowed us to knit the community more closely together with joint seminars, postdoctoral programs, and by funding additional graduate students to be brought into the community. With that support, we went forward and created a new Ph.D. program in Quantum Science and Engineering: the program was accepted by the entire Harvard faculty and approved in the spring of 2021. This is the legacy of our center in creating a unique graduate program that is not physics, electrical engineering, computer science, or applied physics. It’s designed with the recognition that if we are to make some progress in the quantum information system for the future, we need to broaden the expertise, understanding and inclusion of a wide area of engineering and science.

**Figure:**
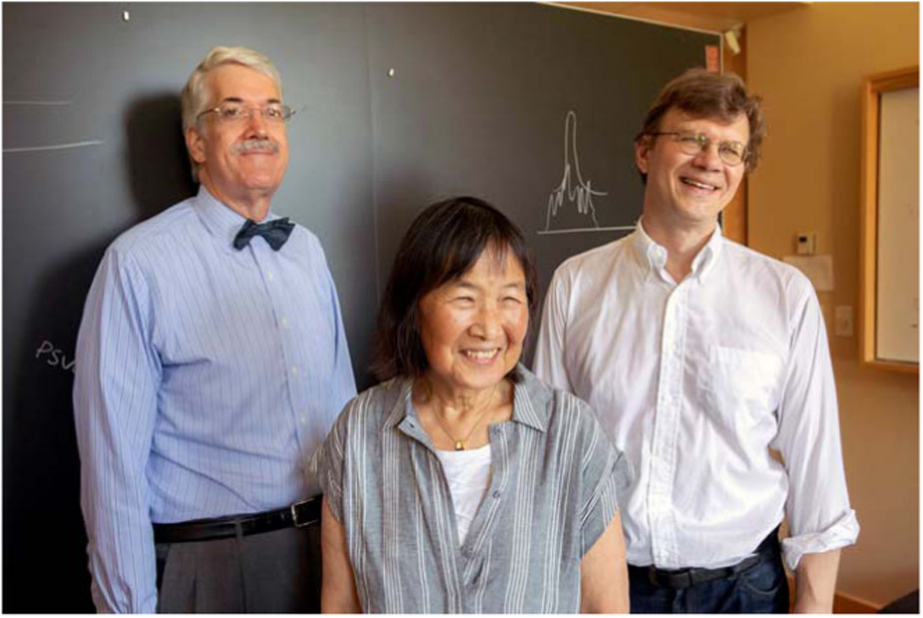
Harvard’s quantum leap (Co-directors of the Quantum Science and Engineering Initiative John Doyle, Evelyn Hu, and Mikhail Lukin.)

**Q10: Where did the seed fund come from?**

**A10:** We got the seed fund from Harvard University with a minimal amount, and then larger amounts from many research grants by NSF, DARPA, Department of Energy, etc. Both types of funds are important. The seed fund from Harvard didn’t really pay for the research to the extent that we needed. But instead, it served as a foundation and a sign, both internally and externally, that “Quantum” is an important emerging intellectual area that Harvard endorses. It gives us the recognition and resources that allow us to better knit the community together.

**Q11: You’re the close disciple of Prof. Chien-Shiung Wu, who was widely called “** ***First Lady of Physics*** **” and “** ***Chinese Marie Curie*** **.” What is her influence on your career? Do you have any memorable moments with her that you’d like to share?**

**A11:** She had a tremendous influence on my career since the time I was very young. Prof. Wu was a hero and a legendary example to my parents, who also came from China to study in the US. Coincidentally, Madame Wu came from a place close to Shanghai, where both my parents grew up. She also spent a large part of her career in Columbia, and I happened to grow up in New York, near Columbia. My parents really hoped that one day I might have a chance to work with her, and it turned out that in fact, I did have that opportunity. So, she did influence my career long before I was old enough to think about a career.

From her parity non-conservation experiment, I can imagine how strongly driven, quick, and energetic she was in her early career. When she heard about the idea from Tsung-Dao Lee and Chen-Ning Yang, she quickly had an idea of how to make the measurement. Though she was limited by the facilities in her own lab, she managed to go to the precursors of NIST and did the work in the lab of Ernest Ambler. I became her student rather late in her career, but her commitment to science was exceedingly strong, and had a strong impact on me. I did my Ph.D. experiment at Brookhaven National Labs. Experiments on accelerators often require a long time to develop and build the instrumentation, to be given time to use the accelerator, and one must be ready to “run” when the schedule is made available for your experiments. The initial setting up of experiments could be extremely time-consuming, and sometimes we would continue with the experiments for times as long as 36 hours, as long as we had access to the accelerator beam. I remember Madame Wu sitting with us during the initial experiments, wanting to be part of the process.

**Q13: What is the initial reason you wanted to pursue a Ph.D. degree in physics?**

**A13:** My parents have always emphasized the importance of higher education ever since my childhood. I see many of my students going through a decision process about pursuing a Ph.D. But I always assumed I would pursue a Ph.D. degree because I loved learning, and I loved continuing my education. The idea of continuing to stay in a learning environment was never a question for me. So, in essence, it wasn’t that I wanted to get the Ph.D. degree; I continued my studies because the Ph.D. was a natural extension of the learning process to me.

**Figure:**
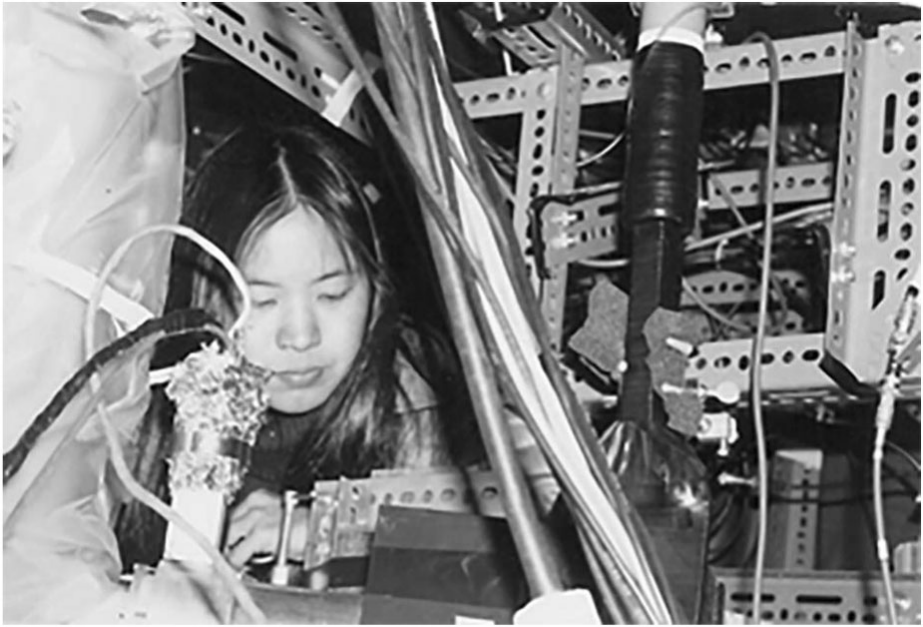
Prof. Hu in her graduate school

**Q15: For many people, even after Ph.D., they will switch to industry or some other career path fundamentally different from science. What made you decide to pursue the faculty track?**

**A15:** Academia is certainly not the only path, perhaps even not the best path for many people. I’ve seen diverse opportunities where different parts of your talents can thrive. You can be even more influential in changing technology and the world in areas besides academia. But for me, the idea of staying in the learning environment and being able to interact with students is the ideal environment. I had always, implicitly assumed that at some level I would go into the university track.

After my Ph.D., I first went to Bell Labs. I was extremely fortunate to be in that wonderful environment with a lot of resources and amazingly bright people expert in a wide range of fields. Although the academic job should have been exactly what I wanted, when I was first offered an academic position, I didn’t feel ready to leave Bell Labs. In fact, it took me a few years to think about what I really wanted before I made the move to UCSB. I think an important motivation to be in academia is that you enjoy teaching and working with students. Otherwise, there are other scientific and technological environments that may be more fulfilling. Now looking back, I am eternally grateful for the opportunity to have worked with the amazing colleagues and friends that I had in Bell Labs. But even so, I felt that there was yet something missing in my complete career satisfaction at Bell Lab. After I made the transition to academia, at UCSB, I realized that the moments of my greatest satisfaction, were linked to contributions I was able to extend to students, and I took great joy in their development and growth: I had found the “something missing”.

**Q16: What is the most important thing that Bell Labs and UCSB left with you?**

**A16:** The most important lessons I learned from both organizations was about the power and fun of scientific collaboration. In Bell Labs, I was fortunate to meet up with Larry Jackel and Rich Howard, who became both outstanding and creative collaborators, as well as good friends.

UCSB provided the same collaborative and strongly supportive environment. When I joined UCSB, it was just beginning to blossom, and had very few instrumental resources. But, more importantly, we had a group of people with a vision. We had James Merz, who did optical characterization. We had Herbert Kroemer, who won the Nobel Prize, and who had one of the first MBE machines in a university. We had Steve Long from Rockwell, who was a gallium arsenide device designer. We had Larry Coldren, who worked in optoelectronics. We brought in Art Gossard, Pierre Petroff, John Bowers and other talented colleagues. We worked together to create powerful and successful new research and education programs. What Bell Labs and UCSB taught me is the power of having people work together, the power of shared vision, and the power of collaboration in research and education. Today, many of the students who went through those programs are now the industrial and faculty leaders in their fields, and they continue to bring the same commitment to collaboration and collegiality. Those are the most important things I learned, which I continue to try to implement.

**Figure:**
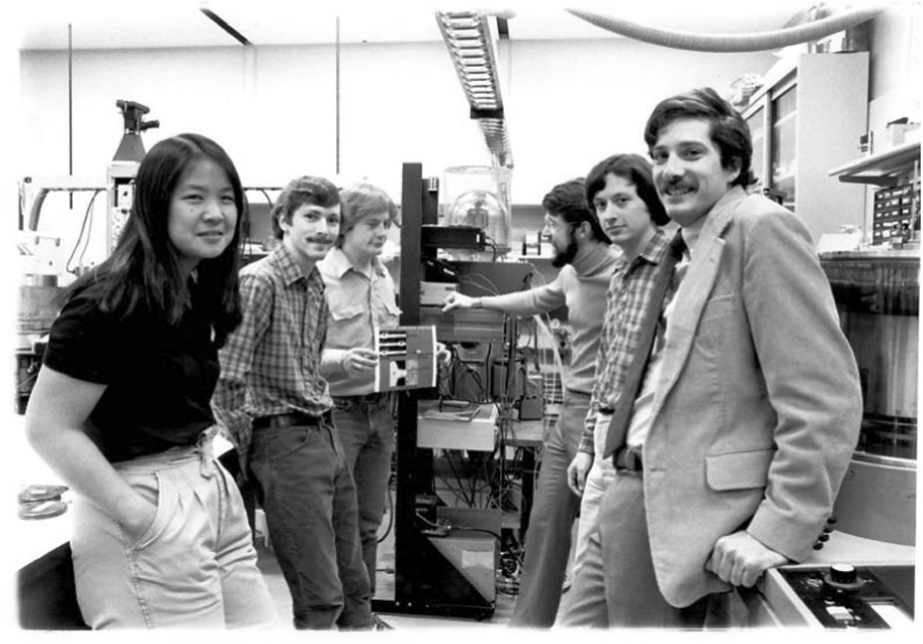
Prof. Hu in Bell labs

**Q17: What is your most memorable moment at UCSB?**

**A17:** So many memorable things that happened at UCSB. Being able to hire people like Umesh Mishra, Arthur Gossard, and Pierre Petroff. Getting the award of the QUEST (Science and Technology Center for Quantized Electronic Structures), one of the first science and technology centers NSF built, was major. I still remember trying to help Jim Merz complete the QuEST proposal, staying up until 4 AM. Creating one of the California Institutes for Science and Innovation, that established the California NanoSystems Institute (CNSI) between UCLA and UCSB. Being able to hire Shuji Nakamura to create one of the strongest academic programs in Gallium Nitride: all a testament to the power of creating a collegial, collaborative community with shared visions and resources.

**Figure:**
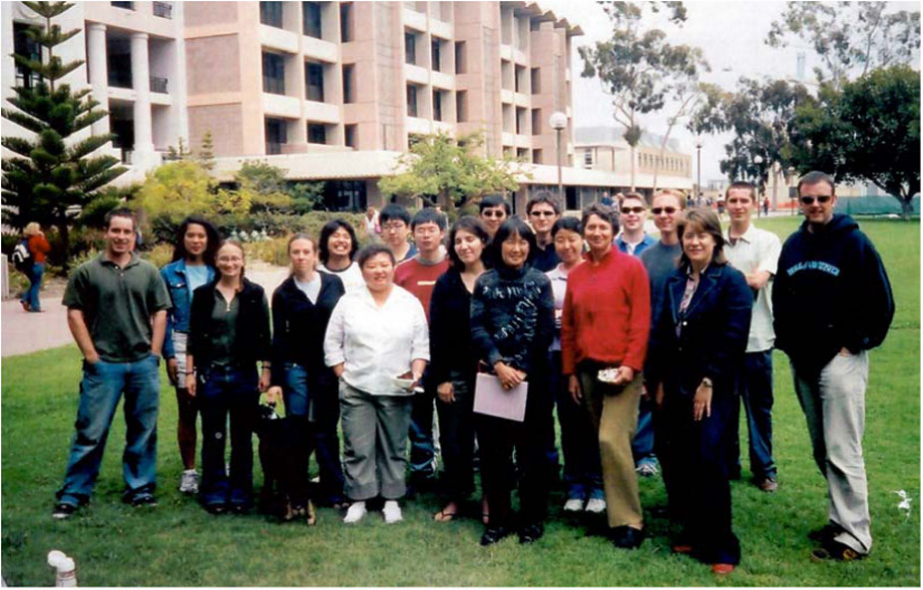
Prof. Hu’s group at UCSB

**Q18: What made you decide to join Harvard?**

**A18:** I had a great love for UCSB, and I’m still very close to my colleagues in UCSB. So why did I leave? I had spent my entire academic career at UCSB, and I thought it was time to try something different; to see what I could learn about myself. There was also a very real concern: my father was aging, and he was in New York. The chance to be closer to him was actually very important at that point.

In addition, when you stay in one place for too long, people start to “type-cast” your activities: the courses you will teach, the roles you will play. When I came to Harvard, the school of engineering and applied sciences was just newly formed and was growing in its undergraduate programs. I had the chance to try teaching a variety of new courses, including courses in applied mathematics. I taught classes of 200 students, as well as smaller courses. So, going to a new place sometimes frees you because you then begin to say: why not try this? Change sometimes releases you from the role that other people think you should fill or the role that you think you should fill. Being in a new place, I had the sense that I could just try more things, and I loved that.

**Figure:**
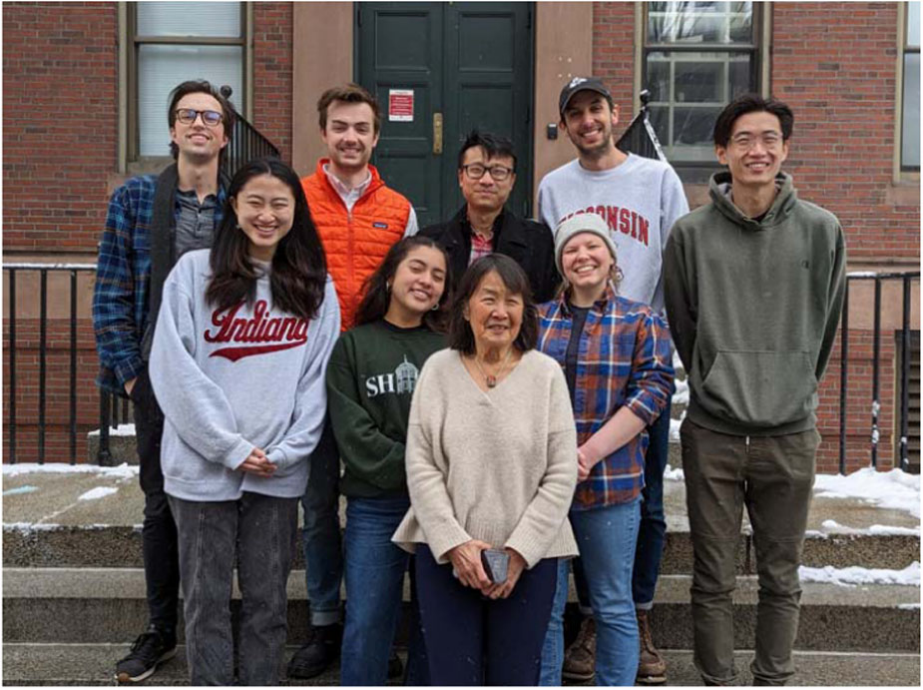
Prof. Hu’s group at Harvard

**Q19: Many students at Harvard named your courses as one of their favorites. How did you manage to get students from different backgrounds to gain interest in your courses?**

**A19:** For the calculus course, there was no issue in attracting students because this is a course almost everybody in sciences needs to take. For the introductory electrical engineering course, there were about 200 people in the class during the time that I taught it. It was an extraordinary electrical introductory class that was called a general education course. This means that not only people who want to study electrical engineering will take it; I also have students from music, history, and government, including both juniors and seniors, that are quite accomplished. I loved that course because I love being able to introduce these exciting concepts to bright people who think they can’t do math or engineering, or they just don’t like it. As part of the course, they also had to do a project and make something electronic. Seeing them just be transformed gives a great sense of accomplishment. So, I got to try widely different courses that attracted students from different backgrounds. I got to redesign and apply electromagnetic courses to include WiFi, radar, antennas, and semiconductor physics. When we had the COVID shutdown, I co-taught a freshman seminar on COVID, from an engineering-science point of view, where the students had a chance to do experiments to measure airflow and mask efficiencies.

**Q20: What’s the difference between teaching undergraduates in your classes and supervising graduates in your lab?**

**A20:** For both situations, the philosophy is always to uncover the magic of the science and the research, to have students appreciate that magic and follow up on it, and to let them discover what they can do themselves with that magic. When teaching a course, you must assess the students in a limited time of 10–14 weeks. Undergraduates are also quite occupied with a lot of other competing demands, sports, clubs, other classes, etc. Therefore, there is not enough time to develop themes or pay enough attention to each student and discover what their strengths are. But when mentoring Ph.D. students, there is plenty of time to develop ideas, to let them discover things, to find what they like or dislike, and to work with each of them over a long period of time.

**Q21: Which do you prefer: in-person courses and online courses?**

**A21:** I think everyone appreciates in-person courses more. When you’re together in a room, you can sense people’s reactions, engage with them, and respond immediately. In situations when I had to teach remotely, it wasn’t easy to have people engaged. Yet, I am really impressed that there are still ways to make it work. When I taught classes remotely, I was really aided by tremendously talented teaching assistants who went to every lecture, and we both utilized icebreakers, create open-ended questions, and really engaged the students in discussions. I even taught a remote class that had a lab component where I was able to find a way to go through the lab component with the students. Remote teaching among a small group of people does have an advantage. You may learn how to communicate with your students more effectively because you really need to have them engaged, and you really need to interact with them so that they feel they are part of a community. I usually asked students about their home environments and had them work on group projects instead of working on individual problem sets. When we had time, I asked students to each give a short description of themselves so that they would share more of who they were; I introduced much more group work with short, open-ended questions to get students to engage with each other and trust each other. I tried to continue that when we went back in person. When they share more about who they are, they engage more with each other, trust each other better, and are more likely to be open to learning.

**Q22: You are a widely appreciated scholar around the world, and for that, you have won numerous awards. What do the awards mean to you? Which one are you most proud of?**

**A22:** I think everyone enjoys being appreciated, so the awards are naturally very meaningful to me. But I’m always aware that if I win an award, that it is a result of my good fortune. There are many other people who are equally deserving. I’m also very grateful to the people who took the time and effort to write nominations for me. Above all, we all need to do what we do without expectations of special awards. Each one of the awards that I received is wonderful, and I can’t pick which one I’m most proud of. But if I just roughly state the categories, the teaching awards are very meaningful to me. Students are rightfully very demanding critics, and I feel it’s always important to work hard to ensure that I am communicating and interacting with them effectively. I was also able to give the second Mildred S. Dresselhaus Lecture. Mildred S. Dresselhaus is a tremendously important female scientist who has donated a tremendous amount to the community and the field. She was a long-time friend and a role model. Being able to give lectures on behalf of Mildred was very meaningful to me, as part of a lecture series established in her honor, because I truly wanted to honor her.

**Q23: What kind of advice would you share with Ph.D. students and postdocs who are pursuing an academic career?**

**A23:** I can provide my advice, but everybody must be always sensitive to their own circumstances. My first piece of advice would be to know what is most important to you and what gives you the greatest satisfaction in your career. The other piece of advice is to really understand what you’re good at, and to give yourself credit for it. This is probably difficult for all of us. We tend to minimize our strengths, and instead, focus on our weaknesses. It is, of course, important to improve. However, we must understand what you do that is distinctly you, and distinctly an advantage. Sometimes you need somebody whom you trust to tell you that. It is important to understand what our strengths are and find situations where our strengths can shine.

**Q24: What advice would you give to the younger version of yourself? Are there any things that you wish you had done differently?**

**A24:** The advice that I would give is to be more daring, to try and push past the limits of comfort more, and to take more risks. Sometimes I wish I had been braver, but that’s advice I can give to myself now, and I’m not sure whether I would follow it.

**Q25: You’re already very brave for always stepping out of your comfort zone and exploring new research areas. This is also very creative. How can we always stay creative? How can we have the courage to step out of our comfort zone?**

**A25:** I don’t have a good answer, for creativity is to be open to ideas. But even so, creativity doesn’t always come. “How to step out of our comfort zone?” Honestly, I am not sure if doing something new after finishing graduate school was brave. I think that I made a change, recognizing that it was necessary for me to stay engaged, excited, and committed to my future career. So, I don’t have any answers except for being open to opportunities and trying different things.

**Q26: What was the most stressful moment in your academic career? And how did you handle stress?**

**A26:** Every day brings its own stress, e.g., paperwork, students having a problem, or breakdowns in the lab. But that’s ordinary stress. I know some people have greater degrees of stress, but I’m so fortunate that I’ve never encountered it. I handle my stress best by running or long-distance walking (several miles) on a frequent basis. Physical activity is one efficient way to get out of tension.

**Q27: How about the stress you face as a female, especially when we must have a balance between work and life?**

**A27:** I’m very fortunate that I haven’t met that stress directly, though I do know female colleagues who have met with discrimination and hostility. I think a big part of it is because of different possible reasons. First, I’m very focused, in good and not-so-good ways. When you focus on a goal, sometimes you just maneuver away from controversy and situations that could have bad outcomes. Second, I have been extremely fortunate to have worked with collaborative groups of people, providing a community that knows me, respects me, and protects me. With the community, I am not isolated; I am not alone, and I have a better ability to work and make an impact. Third, perhaps having a female advisor (Madame Wu in my case), role model aside, saved me from situations that could have been compromising when I was younger and more vulnerable. I, fortunately, navigated a path where I didn’t have to face a lot of those issues which do exist, and which other women may have encountered. But I think things are changing today. I’ve been on important decision-making committees with a larger proportion of female members. It makes a difference, even in the language you use and the feelings you have, participating in more diverse committees and organizations. I do feel more relaxed and freer. In terms of the balance between work and life, I think it’s a matter of growing to know yourself and deciding what’s important to you. If you fully focus on work, you will take all your time away from family and friends. So, you just must understand what’s important and how to adjust for that, although it may not be a perfect balance all the time.

**Q28:** ***Light: Science & Applications*** **has been published for more than a decade. Could you provide a few suggestions for us?**

**A28:** In terms of understanding photonics, science and applications, and engineering of light, I think your journal has a wonderful title. Focus on aspects that may not be covered by other journals. LAS can share with its readership those different aspects and opportunities and grow in that way.

## Supplementary information

Supplementary Video Information

